# Microstructural Characterization of Dry Powder Inhaler Formulations Using Orthogonal Analytical Techniques

**DOI:** 10.1007/s11095-024-03776-1

**Published:** 2024-10-07

**Authors:** Gonçalo Farias, William J. Ganley, Robert Price, Denise S. Conti, Sharad Mangal, Elizabeth Bielski, Bryan Newman, Jagdeep Shur

**Affiliations:** 1 Pharmaceutical Surface Science Research Group, Department of Pharmacy & Pharmacology, University of Bath, Bath, UK; 2 Nanopharm Ltd, an Aptar Pharma Company, Wales, UK; 3 Office of Research and Standards, Office of Generic Drugs, Center for Drug Evaluation and Research, Food and Drug Administration, Silver Spring, MD USA

**Keywords:** bioequivalence, dissolution, dry powder inhaler, orthogonal analytical techniques, raman spectroscopy

## Abstract

**Purpose:**

For locally-acting dry powder inhalers (DPIs), developing novel analytical tools that are able to evaluate the state of aggregation may provide a better understanding of the impact of material properties and processing parameters on the *in* *vivo* performance. This study explored the utility of the Morphologically-Directed Raman Spectroscopy (MDRS) and dissolution as orthogonal techniques to assess microstructural equivalence of the aerosolized dose of DPIs collected with an aerosol collection device.

**Methods:**

Commercial DPIs containing different strengths of Fluticasone Propionate (FP) and Salmeterol Xinafoate (SX) as monotherapy and combination products were sourced from different regions. These inhalers were compared with aerodynamic particle size distribution (APSD), dissolution, and MDRS studies.

**Results:**

APSD testing alone might not be able to explain differences reported elsewhere in *in* *vivo* studies of commercial FP/SX drug products with different Advair® strengths and/or batches. Dissolution studies demonstrated different dissolution rates between Seretide™ 100/50 and Advair® 100/50, whereas Flixotide™ 100 and Flovent® 100 had similar dissolution rates between each other. These differences in dissolution profiles were supported by MDRS results: the dissolution rate is increased if the fraction of FP associated with high soluble components is increased. Principle component analysis was used to identify the agglomerate classes that better discriminate different products.

**Conclusions:**

MDRS and dissolution studies of the aerosolized dose of DPIs were successfully used as orthogonal techniques. This study highlights the importance of further assessing *in* *vitro* tools that are able to provide a bridge between material attributes or process parameters and *in* *vivo* performance.

## Introduction

Complex formulation/device combinations performance, for instance dry powder inhalers (DPIs), depends on multiple factors such as patient/device and formulation/device interactions, manufacturing, and processing procedures. The extent of this complexity has been illustrated by a recent study showing that multiple batches of the same DPI product can contain batch-to-batch variability based on systemic exposure via pharmacokinetic (PK) data [1, 2]. Thus, it is important to understand the critical performance attributes of the DPI product as well as patient factors in order to achieve consistency and equivalence in product performance and systemic PK.

The magnitude of the scientific challenges can be highlighted by the complexity of a carrier-based DPI drug product. Since a formulated blend does not attain thermodynamic equilibrium, the resultant formulation structure is dependent on the relationship between material properties and processing parameters [3]. These include a series of critical material attributes, environmental conditions, critical manufacturing processes, and conditions of storage [4, 5]. Discrepancies in the microstructural arrangement of DPI formulations could alter the aerosolization performance of a DPI device, and consequently its site of delivery within the lungs [6]. Therefore, it remains critical for DPIs to evaluate the state of aggregation pre- and post-aerosolization if attempting to demonstrate microstructural similarity and equivalent product performance between DPI products. Microstructural characterization involves analysis of the aggregated state and particle interactions. To achieve understanding in product performance, characterization of DPI products requires an ensemble of orthogonal techniques that are both structurally sensitive, are complementary to each other and have a chemical resolution due to the complexity of these products. As such, the assessment of the dissolution profile and state of agglomeration via Raman Spectroscopy of the aerosolized fraction of DPIs has emerged as potential techniques.

Dissolution of low soluble compounds is sensitive to their microstructure (e.g. size, morphology and state of agglomeration) due to a dependence on their “wettable” surface area. This can be increased by agglomeration with a more soluble component (such as lactose) which dissolves rapidly, leaving drug particles with a higher “wettable” surface area, as previously demonstrated by Nyström and Westerberg (1986) for griseofulvin ordered mixtures [7]. Previous dissolution studies of micronized drugs in interactive mixtures have also shown that dissolution rates were concentration-dependent with increasing drug concentration, increasing the percentage of hydrophobic agglomerates and decreasing the dissolution rate [8–10].

Accompanying chemical composition analysis of the aerosolized agglomerate particles, together with dissolution studies, may help to probe the microstructure of DPI drug products. Chemical discrimination of agglomerate structures requires molecular rather than elemental specificity, as many pharmaceutical materials are comprised of similar elements. Raman spectroscopy, in particular, has been used extensively in the analysis of tablets [11, 12], drug-eluting stent formulations [13] and particles aerosolized from inhalers [14–16] to observe both drug and excipient distribution with a resolution of a few microns and has been used to track chemical changes occurring during tablet dissolution [17, 18]. Spectroscopic determination of molecules is a powerful technique as the information from full spectra can be combined using multivariate statistical techniques [19] to identify molecules even when their Raman bands overlap [20, 21]. Techniq﻿ues such as MDRS are able to identify the chemical nature of aerosolized aggregates. MDRS has previously been used to identify the association between budesonide and fine lactose collected from stage 2 of an Next Generation Impactor (NGI) providing evidence that the increase in fine particle delivery upon the addition of lactose fines is due to aggregate formation [15].

The aim of this study was to explore the *in* *vitro* dissolution and MDRS as orthogonal techniques for the analysis of the impactor-sized mass (ISM) dose of DPI products using an aerosol collection device. A relationship between the extent of agglomeration and dissolution kinetics in RLD products Seretide™ Accuhaler™, Advair® Diskus®, Flixotide™ Accuhaler™ and Flovent® Diskus® DPIs is investigated, and multivariate statistical methods are used to differentiate products of different type (single and combination product), different dose (100, 250 and 500 µg of fluticasone propionate) and different territory (fixed dose and type but marketed either in Europe [EU] or the United States [US]).

## Materials and Methods

### Commercial Dry Powder Inhaler (DPI) Selection and Characterization

The following commercial dry powder inhalers (GlaxoSmithKline, Ware, UK) were procured and included in the study: (i) fluticasone propionate and salmeterol xinafoate (FP/SX) combination therapy – Advair® Diskus® 100/50 (lot 5ZP1526, exp: 02/2017), 250/50 (lot 6ZP4158, exp 07/2017) and 500/50 (lot 6ZP4628, exp 09/2018) and Seretide™ Accuhaler™ 100/50 (lot L98F, exp: 09/2017), and (ii) fluticasone propionate (FP) monotherapy – Flixotide™ Accuhaler™ 100 (lot AE4R, exp: 03/2017) and Flovent® Diskus® 100 (lot 5ZP9260, exp: 09/2016). These batches were used for all tests in the current *in* *vitro* study.

*In* *vitro* aerosolization performance was measured using a NGI equipped with pre-separator (Copley Scientific, Nottingham, UK) and United States Pharmacopeia (USP) induction port. One dose (e.g., one actuation from one blister) from each inhaler was aerosolized at a fixed flow rate 60 L min^−1^ with a duration of 4.0 s, which is expected to be used to characterize this product [22]. Each determination was performed in triplicate. Chemical analyses of the APIs were conducted using a validated HPLC method described previously elsewhere [23].

After quantifying the API from each impactor stage, different parameters were derived: ISM, percent fine particle fraction below 5 µm (%FPF < 5 µm), mass median aerodynamic diameter (MMAD) and geometric standard deviation (GSD). While the ISM corresponds to the sum of mass from stage 2 to stage 8, the %FPF < 5 µm, MMAD, and GSD can be extrapolated from the cumulative drug mass *versus* stage cut-off size plot. The MMAD corresponds to the average particle size of the material collected from the impactor stages and the GSD the width of the distribution [24].

### Aerosol Collection Apparatus (ADC) of the Impactor-Sized Mass (ISM) Dose

An ADC that enables uniform deposition of the whole ISM dose onto a single high surface area 47 mm Pall A/E type glass fiber filter (Copley Scientific, Nottingham, UK) under laminar flow and an order of magnitude lower impaction velocity was used. The development and validation of this aerosol collection apparatus are discussed elsewhere [23].

Commercial DPI formulations were aerosolized into the ADC system via a USP inlet or a coated (brij-35:glycerol:ethanol:Milli-Q water, 1.7:38:54:6.3% v/v) medium Oropharyngeal Consortium (OPC) realistic mouth-throat model (Emmace Consulting, Lund, Sweden) attached to a pre-separator and NGI at a fixed flow rate of 60 L min^−1^ for 4.0 s and collected onto a 47 mm Pall A/E type glass fiber filter (Copley Scientific, Nottingham, UK).

### Dissolution Studies

Dissolution studies of the ISM were conducted in a modified USP paddle over disk (POD) Apparatus V (Erweka GmbH, DT 126, Heusenstamm, Germany), which was altered to accommodate a 47 mm glass fiber filter [25].

To assess the dissolution behavior of non-aerosolized formulation, samples were taken directly from the 6-unit dose blisters. A mass of 12.5 mg was pre-weighed and sieved using a 74 mesh (250 µm) stainless steel screen directly into a USP Apparatus II.

For both the aerosolized and non-aerosolized formulation, a dissolution media composed of 300 mL phosphate buffered saline (PBS) containing 0.2% w/v sodium dodecyl sulfate (SDS) was kept at 37ºC with a stirring speed of 75 rpm. The dissolution of FP was evaluated at different timepoints (2.5, 5, 10, 15, 20, 25, 30, 60, 120, 180, and 240 min) by collecting 3 mL aliquots, which were filtered with a 0.2 µm PTFE syringe filter directly into HPLC vials and the volume withdrawn was replaced with pre-warmed dissolution media. At each time point, the percentage of the drug dissolved was determined by dividing the amount of dissolved drug by the amount dissolved drug after 240 min. All dissolution studies were conducted under sink conditions [23]. Herein, the focus for dissolution studies was on fluticasone propionate. The HPLC method used for FP quantification is described elsewhere [23]. Since salmeterol is not dissolution limited, and 100% of the dose was dissolved by the first-time point (data not shown), SX was not evaluated for dissolution analysis [26].

The dissolution profiles were compared with two methods: a first-order drug release model, which was used to calculate the dissolution half-life (t_0.5_) and the dissolution rate (k_1_), and a model-independent method via calculation of the mean dissolution time (MDT) [27].

### Raman Spectroscopy of DPI Particle Structures Post-Aerosolization

The collected ISM dose using the ADC apparatus was also utilized for MDRS. Upon collection, the filter substrate was mounted on to the sample stage of a Morphologi G3-ID® automated image analysis and Raman Chemical Imaging system (Malvern Panalytical, Worcestershire, UK).

The ADC apparatus previously discussed was used to collect the aerosolised ISM dose onto a filter. The ADC was positioned under stage 2 of the impactor since, unlike stage 1, it has a higher cut-off and captures particles below 4.46 µm at 60 L min^−1^ [24]. The DPI drug products were actuated at a fixed flow rate of 60 L min^−1^ for 4.0 s (equivalent to 4 L of inhaled volume). Forty-seven millimeter cellulose acetate filters (Sartorius, Goettingen, Germany) were used for this analysis to allow the deposition of the particles onto a single layer to be easily detected under a microscope. One actuation was used since it was enough to get a representative number of particles to be detected under the microscope without any induced agglomeration.

The imaging parameters on the MDRS (e.g. threshold and light settings) were optimized to ensure an appropriate identification of aerosolized particles and its full perimeter. Diascopic bottom light with 70% intensity ensured good contrast between particles and filter and a corresponding threshold of 155 on a greyscale provided delimitation and identification of the particles with reduced background. Since the particles being analyzed have an aerodynamic diameter lower than 4.46 µm, the highest magnification (50x) was selected as it is able to capture particles between 0.5 to 50 µm. No filters were applied at this stage to enhance the efficiency of the system.

A reference spectra library of FP, SX, and lactose monohydrate (Lac) was built. The spectra range of 325–512 cm^−1^, 700–765 cm^−1^, 970–1055 cm^−1^, 1174–1226 cm^−1^ and 1554–1695 cm^−1^ were selected for comparison to focus the analysis on the main identifiable peaks for the various chemical identities. To further enhance the chemical identification by reducing the noise in the spectrum, the background spectra for each measurement was subtracted and an intermediate smoothing (Savitsky-Golay filtering over 31 points) of a second derivative was applied [28].

The reference library was then used for identifying the chemical composition of the particles collected on the aerosolized sample, which were classified into different chemical classes according to its correlation score against the reference spectra. The correlation score thresholds selected are presented in Table I and are higher for the individual chemical identities than when these are agglomerated. A laser time of five seconds of spectrometry acquisition per particle was chosen since a dry sample was tested, and no major impact was observed when the laser time was reduced.

**Table I:** Correlation score limits for the different single compound and aggregate classes

Chemical Identity	Single compound thresholds	Agglomerates thresholds
Fluticasone Propionate	0.50	0.35
Salmeterol Xinafoate	0.40	0.12
Lactose Monohydrate	0.50	0.19

**Fig. 1:**
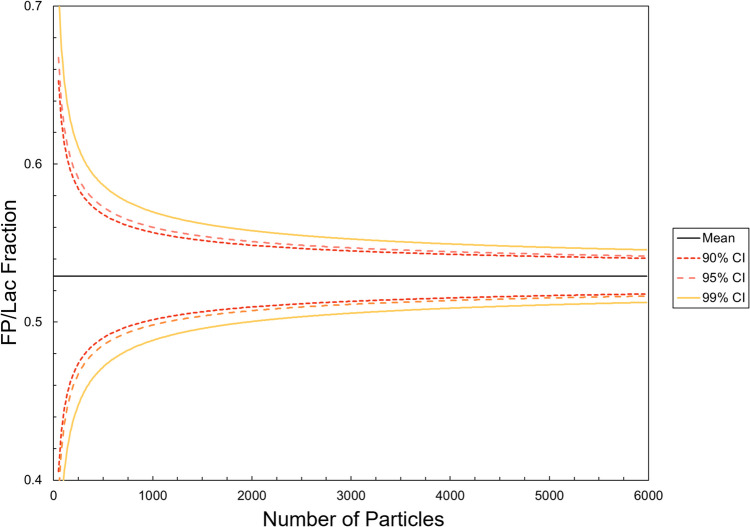
Confidence intervals of the sampling distribution for FP/Lac.

The scan area was set to 4.5 mm by 4.5 mm, and the minimum number of particles per repetition was selected after analyzing around 23,000 particles from six individual MDRS runs of Advair® Diskus® 100/50. This scan area was positioned on a random location of the filter after assessing that the results in different areas of the filter (top, center, bottom, left, and right) were similar. To estimate how many particles would be required to get statistically meaningful data, the main chemical class was selected: FP/Lac. This class is also expected to be the most meaningful for this study since its focus was on using MDRS as an orthogonal technique to dissolution and understanding how the agglomeration of a low solubility API (FP) with lactose impacted its dissolution [7]. Therefore, confidence intervals around the proportion of FP/Lac in a sample were estimated to determine the number of particle spectra required for accurate measurement of the chemical class fractions. The classification of a particle as an FP/Lac agglomerate was treated as a binomially distributed variable, and confidence intervals around its proportion were estimated using the normal approximation (which is valid at large sample sizes). Using a population mean estimate of 0.53 (equal to the proportion of FP/Lac particles in the large, pooled sample), the 99% confidence interval width fell below 10% of this mean after around 3000 particle spectra were analyzed (Fig. 1). In the experiments described in the rest of the study, more than 3000 particle spectra were analysed in each individual MDRS experiment.

### Statistical Analysis

Statistical analysis and principal component analysis were carried out using R version 3.3.1 software. Probability values of < 0.05 were considered as statistically significant. The Shapiro–Wilk test was used to check for normality (α = 0.05) prior to carrying out t-tests, and the assumptions of the analysis of variance (ANOVA) model were checked visually using the diagnostic plots output by R. No violations of any test or model assumptions were found.

## Results and Discussion

### Aerodynamic Particle Size Distribution of DPI Products

**Fig. 2:**
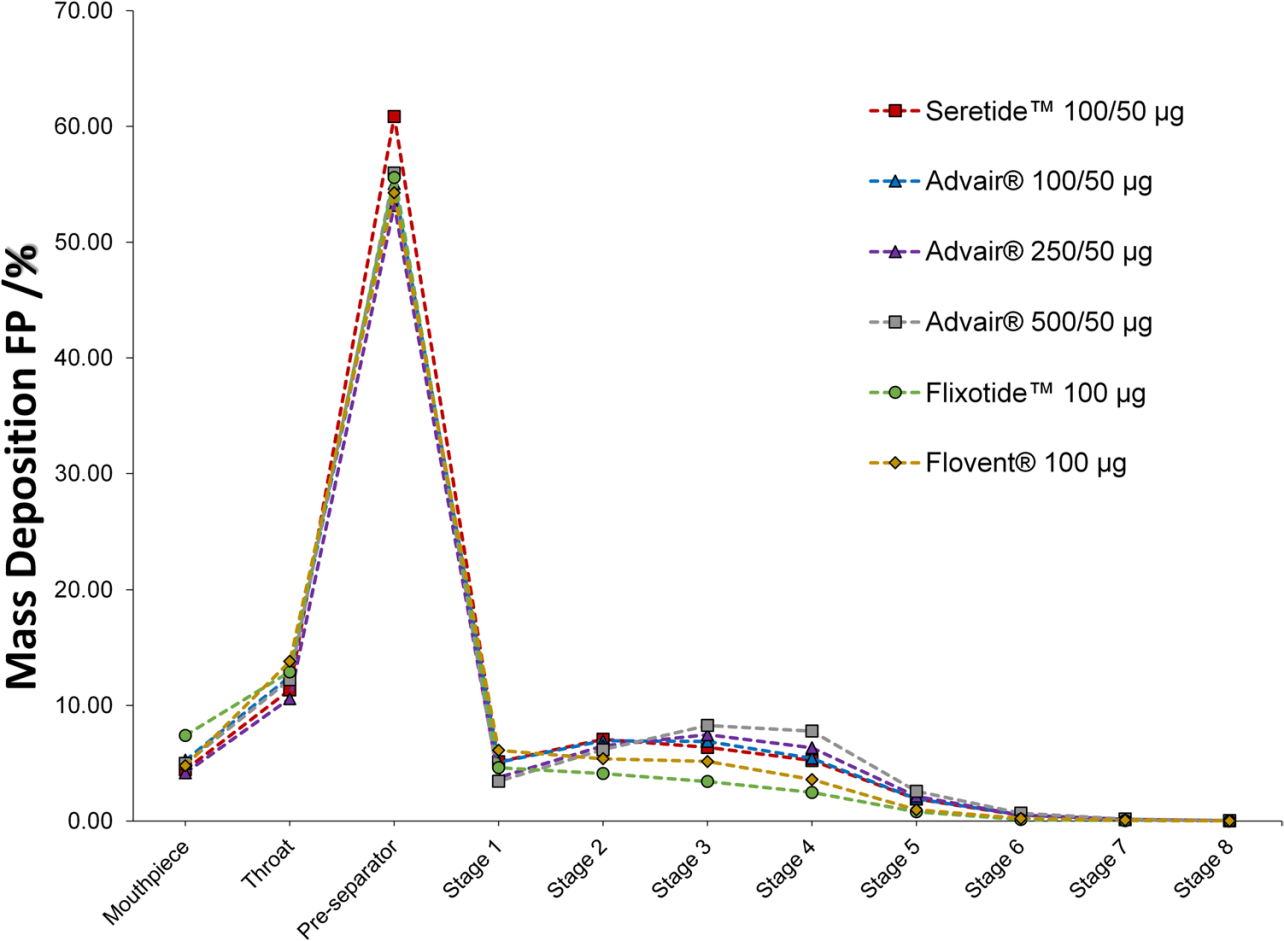
Normalized mean APSD profiles of FP against delivered dose label claim at 60 L/min for Seretide™ Accuhaler™100/50, Advair® Diskus® 100/50, Advair® Diskus® 250/50, Advair® Diskus® 500/50, Flixotide™ Accuhaler™ 100 and Flovent® Diskus.® 100.

As a common critical quality attribute for dry powder inhalers and compendial methods, the APSD of all DPI batches studied in the current study has been investigated. APSD results shown in Table II and Fig. 2, suggest that there is no statistical differences (*p* < 0.05) for ISM fraction, %FPF < 5 µm, MMAD and GSD for Seretide™ Accuhaler™ 100/50, Advair® Diskus® 100/50 and Advair® Diskus® 250/50.

**Table II:** The range of values for ISM fraction and FPF as a percentage of the delivered dose label claim***,*** MMAD (µm) and GSD of FP for Seretide™ Accuhaler™100/50, Advair® Diskus® 100/50, Advair® Diskus® 250/50, Advair® Diskus® 500/50, Flixotide™ Accuhaler™ 100 and Flovent® Diskus® 100 tested at 60 L/min

Product	ISM (%)	FPF < 5 µm (%)	MMAD (µm)	GSD
Seretide™ Accuhaler™100/50	17.3 – 22.0	12.1 – 16.9	3.8 – 4.6	2.0 – 2.1
Advair® Diskus® 100/50	18.9 – 21.8	14.0 – 16.4	3.9 – 4.2	2.0 – 2.0
Advair® Diskus® 250/50	20.2 – 23.1	15.8 – 18.6	3.5 – 3.9	2.0 – 2.1
Advair® Diskus® 500/50	23.5 – 24.7	19.1 – 20.2	3.4 – 3.4	2.0 – 2.1
Flixotide™ Accuhaler™ 100	9.8 – 11.5	6.9 – 8.1	4.7 – 5.0	2.1 – 2.2
Flovent® Diskus® 100	12.4 – 16.1	8.9 – 11.8	3.8 – 4.6	2.1 – 2.5

A comparison of literature systemic PK profiles of FP following two-doses from Advair® Diskus® 100/50 (total dose of 200 µg of FP) *versus* a single dose from Advair® Diskus® 250/50 show large differences in PK profiles, which might not be simply justified by the slight difference in emitted dose of FP [29]. This issue is compounded when searching for PK studies where inter-batch variability of DPI commercial formulations has been found to be large [1, 2]. This may be explained by the difficulty in achieving consistent DPI products in different batches with identical microstructure (i.e., agglomeration state before and after aerosolisation) owing to the high level of complexity during the manufacturing process of these products including the raw material properties, processing parameters, and environmental conditions. Thus, the possibility of identifying differences between two or more separately manufactured lots by using advanced *in* *vitro* characterisation techniques, such as dissolution of the aerosolized dose (ISM) and Raman spectroscopy, to characterise a DPI product could be beneficial, e.g., batch-to-batch or generic to reference product equivalence.

### *InVitro* Dissolution of ISM Dose of DPI Products

In order to provide an improved way to sample DPIs for dissolution studies, which have the potential to provide bridging information between local drug deposition and systemic absorption through the lung, we developed an ADC apparatus [30]. The system was originally conceived to evaluate the dissolution rate of DPIs by capturing the whole ISM dose uniformly into a filter [23]. The use of this aerosol collection method enabled the dissolution release profiles to be measured in a dose independent, which significantly ameliorated the robustness and sensitivity of the methodology [23, 31]. This ADC apparatus is also being explored to evaluate the drug release and permeation of highly soluble drug substances [32].

**Fig. 3:**
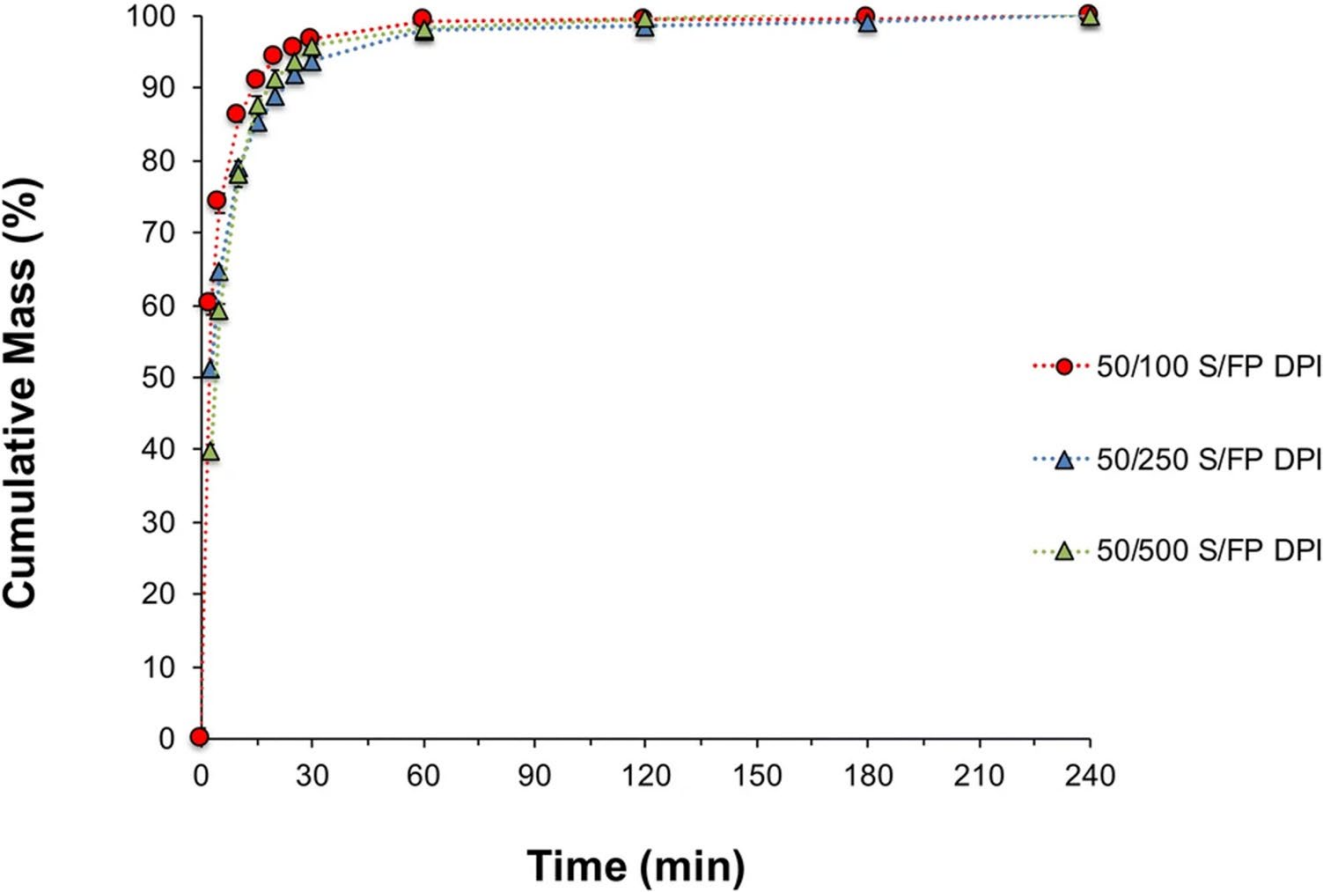
Mean cumulative mass (%) dissolution profiles of the FP ISM dose of Seretide™ Accuhaler™ 100/50, 250/50 and 500/50, DPIs collected at 60L/min with a USP inlet and pre-separator connected to the ADC apparatus. Error bars show standard deviations of 3 repeated measurements. Data previously published [23].

The *in* *vitro* dissolution profiles of the FP component of the filter-borne aerosolized ISM from Seretide™ Accuhaler™ 100/50, 250/50, and 500/50 FP/SX (µg/µg) with different batches to the ones herein used has been discussed by our group in a previous study [23]. In this study, presented in Fig. 3, the dissolution rate of the FP dose was inversely proportional to drug loading, in which the low strength exhibited the fastest rate of dissolution and the high strength product the slowest rate of dissolution. Although these products had an equivalent concentration of SX and blend fill weight, the increase of FP concentration in these unit dose carrier-based blister formulations, resulted in a reduced dissolution rate of the aerosolized drug dose. These findings for DPI formulations are supported by previous studies for oral and topical drug products that demonstrated that an increasing surface coverage of poorly soluble compounds in blends, decreased the dissolution rate of these molecules [7, 33].

**Fig. 4:**
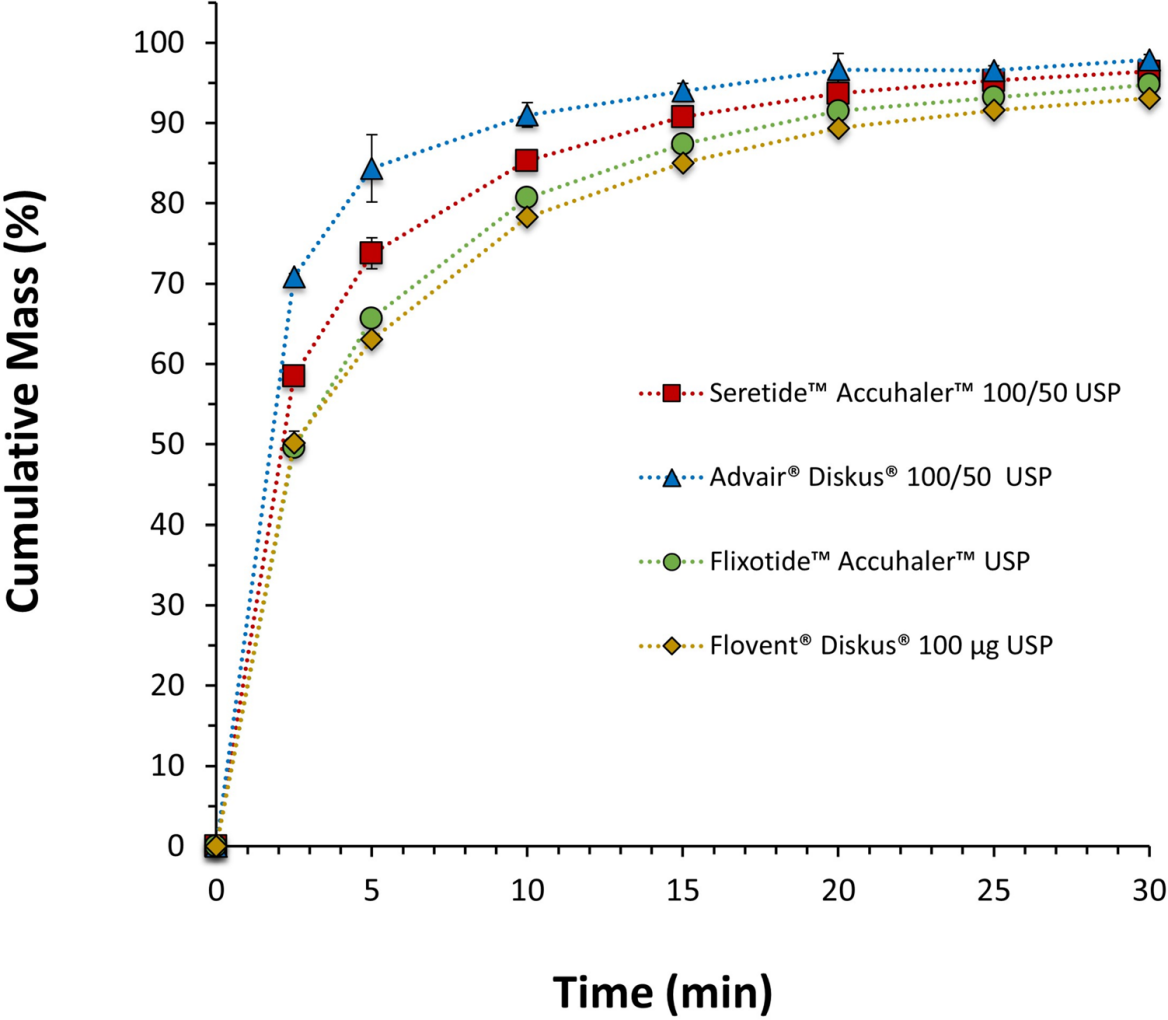
Mean cumulative mass (%) dissolution profiles up to 30 min (zoomed in from 120 min) of the FP ISM dose of Seretide™ Accuhaler™ 100/50, Advair® Diskus® 100/50, Flixotide™ Accuhaler™ 100 and Flovent® Diskus® 100, DPIs collected at 60L/min with a USP inlet and pre-separator connected to the ADC apparatus. Error bars show standard deviations of 3 repeated measurements.

The dissolution of the FP component of the aerosolized ISM dose with the ADC apparatus coupled with a pre-separator and USP throat of Flixotide™ Accuhaler™ 100, Flovent® Diskus® 100, Seretide™ Accuhaler™ 100/50 and Advair® Diskus® 100/50 is shown in Fig. 4. The dissolution kinetics of the different products are summarized in Table III. All products have a fixed concentration of FP and a constant blend fill weight (12.5 mg), two of them have SX (Seretide™ Accuhaler™ and Advair® Diskus®), and each pair of products was sourced from two different origins: US (Flovent® Diskus® and Advair® Diskus®) and EU (Flixotide™ Accuhaler™ and Seretide™ Accuhaler™). For the monotherapy drug products, sourcing them from different territories did not impact the dissolution rate. Interestingly, the same was not observed for the combined therapy products (Fig. 4 and Table III). Seretide™ Accuhaler™ had a slower dissolution rate for FP compared to Advair® Diskus® even though these products are manufactured from the same company and contain qualitative and quantitative similar formulations. These observations were supported by dissolution profile comparison results (first-order release and MDT) presented in Table III. The presence of a highly soluble drug, such as SX, also enhanced the dissolution rate of FP in the combined therapy products, as reported elsewhere [23].

**Table III:** Dissolution rate of FP post-aerosolization (*n* = 3, mean ± standard deviation) for DPI products containing FP using the USP inlet and pre-separator connected to the ADC apparatus sampling method (ISM USP) or the medium-sized OPC throat and pre-separator connected to the ADC apparatus sampling method (ISM OPC), and dissolution rate of FP from the bulk powder (without aerosolization)

Product	Dose Sample	k_1_ (min^−1^)	T_0.5_ (min)	MDT (min)
Flixotide™ Accuhaler™100 EU Brand FP DPI	ISM USP	0.110 ± 0.001	6.32 ± 0.12	4.98 ± 0.34
	ISM OPC	0.109 ± 0.008	6.32 ± 0.35	5.02 ± 0.59
	Powder	0.110 ± 0.003	6.32 ± 0.15	4.58 ± 0.16
Flovent® Diskus® 100 US Brand FP DPI	ISM USP	0.100 ± 0.012	7.01 ± 0.11	5.12 ± 0.48
	ISM OPC	0.099 ± 0.002	6.98 ± 0.15	5.02 ± 0.30
	Powder	0.115 ± 0.002	6.00 ± 0.09	4.48 ± 0.15
Seretide™ Accuhaler™ 100/50 EU Brand FP/SX DPI	ISM USP	0.138 ± 0.030	5.03 ± 0.16	4.11 ± 0.18
	ISM OPC	0.137 ± 0.005	5.06 ± 0.17	4.02 ± 0.05
	Powder	0.141 ± 0.002	4.93 ± 0.08	3.28 ± 0.04
Advair® Diskus® 100/50 US Brand FP/SX DPI	ISM USP	0.194 ± 0.009	3.57 ± 0.26	3.12 ± 0.13
	ISM OPC	0.177 ± 0.006	4.06 ± 0.21	3.53 ± 0.09
	Powder	0.203 ± 0.010	3.41 ± 0.26	2.52 ± 0.07

**Fig. 5:**
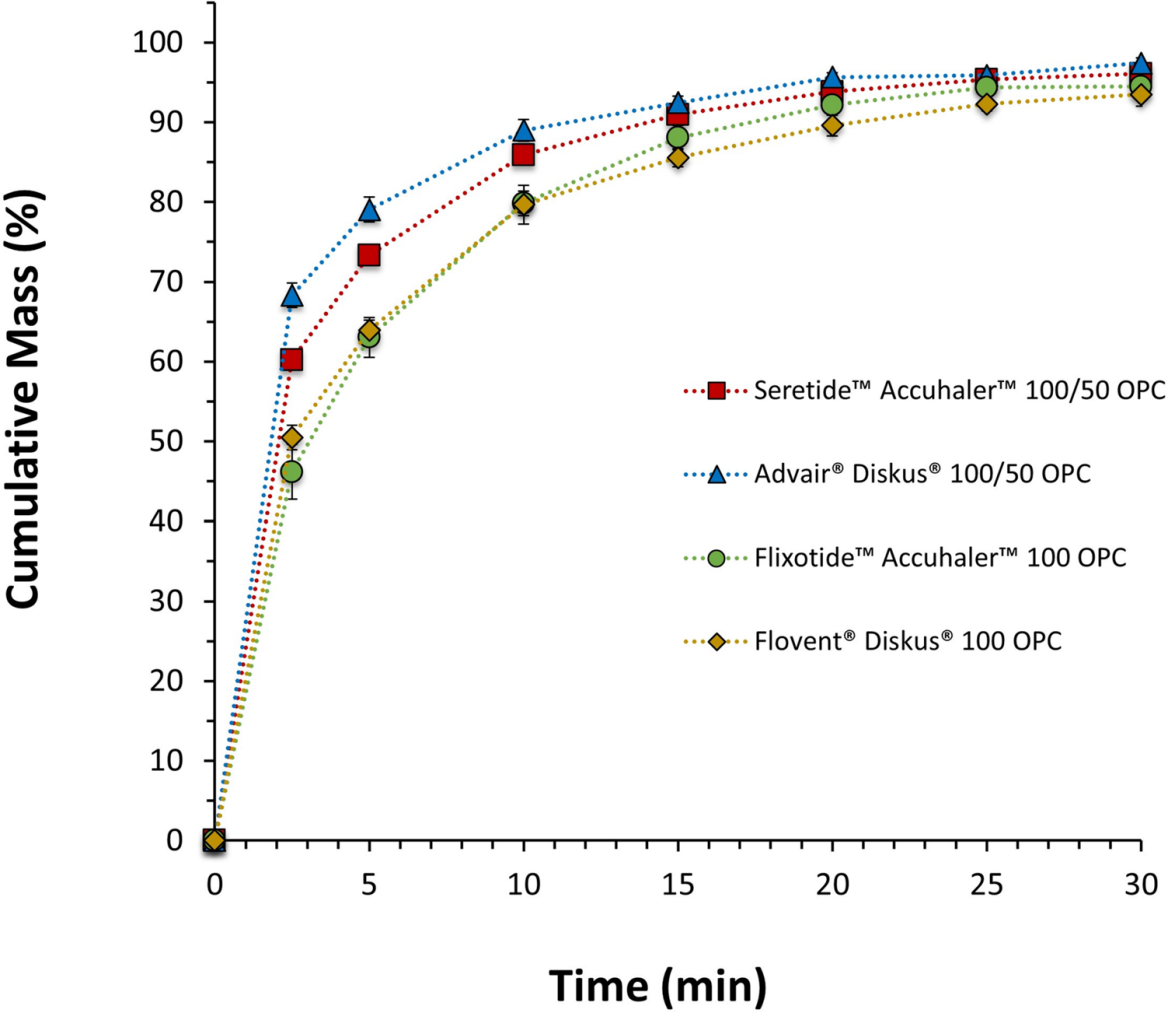
Mean cumulative mass (%) dissolution profiles up to 30 min (zoomed in from 120 min) of the FP ISM dose of Seretide™ Accuhaler™100/50, Advair® Diskus®100/50, Flixotide™ Accuhaler™100 and Flovent® Diskus® 100, DPIs collected at 60 L min^−1^ with a medium-sized OPC throat and pre-separator connected to the ADC apparatus. Error bars show standard deviations of 3 repeated measurements.

The ISM dissolution of the same commercial products was also evaluated with a more realistic throat (OPC throat) as presented in Fig. 5. The same trends as for the USP inlet were observed as summarized in Table III.

**Fig. 6:**
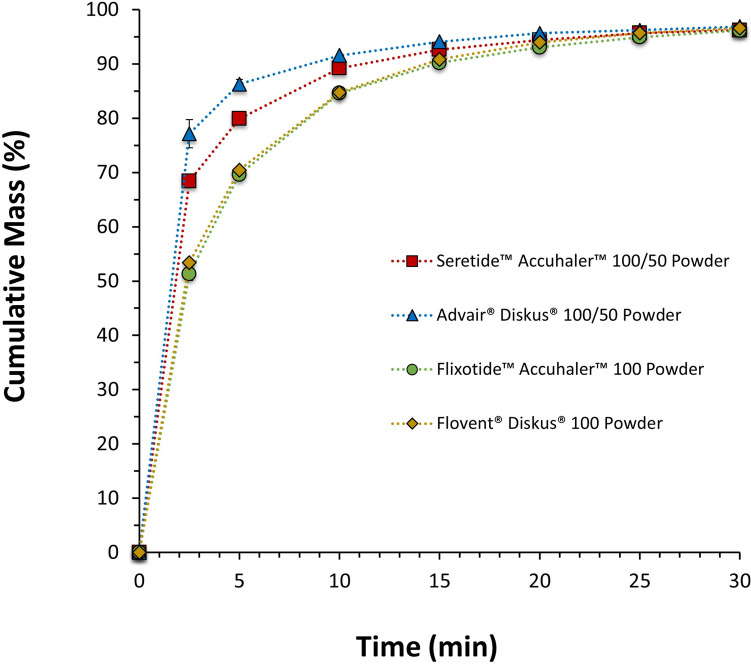
Mean cumulative mass (%) dissolution profiles up to 30 min (zoomed in from 120 min) of the FP formulated powder of Seretide™ Accuhaler™ 100/50, Advair® Diskus® 100/50, Flixotide™ Accuhaler™ 100 and Flovent® Diskus® 100, DPIs. Error bars show standard deviations of 3 repeated measurements.

The observed differences in the dissolution rate of FP post-aerosolization could potentially be explained by differences in the microstructure of the powder blend. To assess this hypothesis, the dissolution of all commercial products powder without aerosolization was evaluated by weighing 12.5 mg of the powder inside each blister cavity and sieving into a USP II apparatus as shown in Fig. 6. The dissolution kinetics of these powders followed the same trend as the aerosolized material, and, once again, only the dissolution profiles of the monotherapy products was assessed to be similar. Whilst the cause of these difference between products sourced from different territories remains unclear, these are probably linked to differences in the microstructure of the products, which could be a result of batch-to-batch variability, physicochemical properties of the raw materials, and intrinsic factors involved on the production of these batches such as manufacturing factors, transport, age and storage.

These differences in the rate of dissolution of the poorly soluble FP from products obtained in different territories, as well as dissolution differences between dose strengths of FP/SX fixed combination products, suggest that the local interparticle interactions within each drug product may not only dominate aerosol performance but also influence drug dissolution within the lung. Previous studies in interactive mixtures of solid oral dosage forms have demonstrated that the dissolution rate of low solubility drugs depends on its degree of dispersion in more soluble excipients [34]. To further investigate this premise, the agglomeration state of FP ISM dose was evaluated by MDRS.

### Microstructure of ISM Dose of the DPI Products Using Raman Microscopy

To elucidate the relationship between powder structure (e.g., API-API/API-excipient interactions and agglomeration behavior) and *in* *vitro* dissolution, some form of high-resolution microscopy is needed. The recent combination of morphologically-directed microscopy and chemical identification via Raman spectroscopy has enabled both drug- and excipient-specific data to be obtained within multi-component systems such as nasal suspension formulations [35]. The use of MDRS to spatially map the state of aggregation of API and excipients of the aerosolized dose from DPIs is, therefore, an interesting potential method for evaluating both mono and combination formulations.

**Fig. 7:**
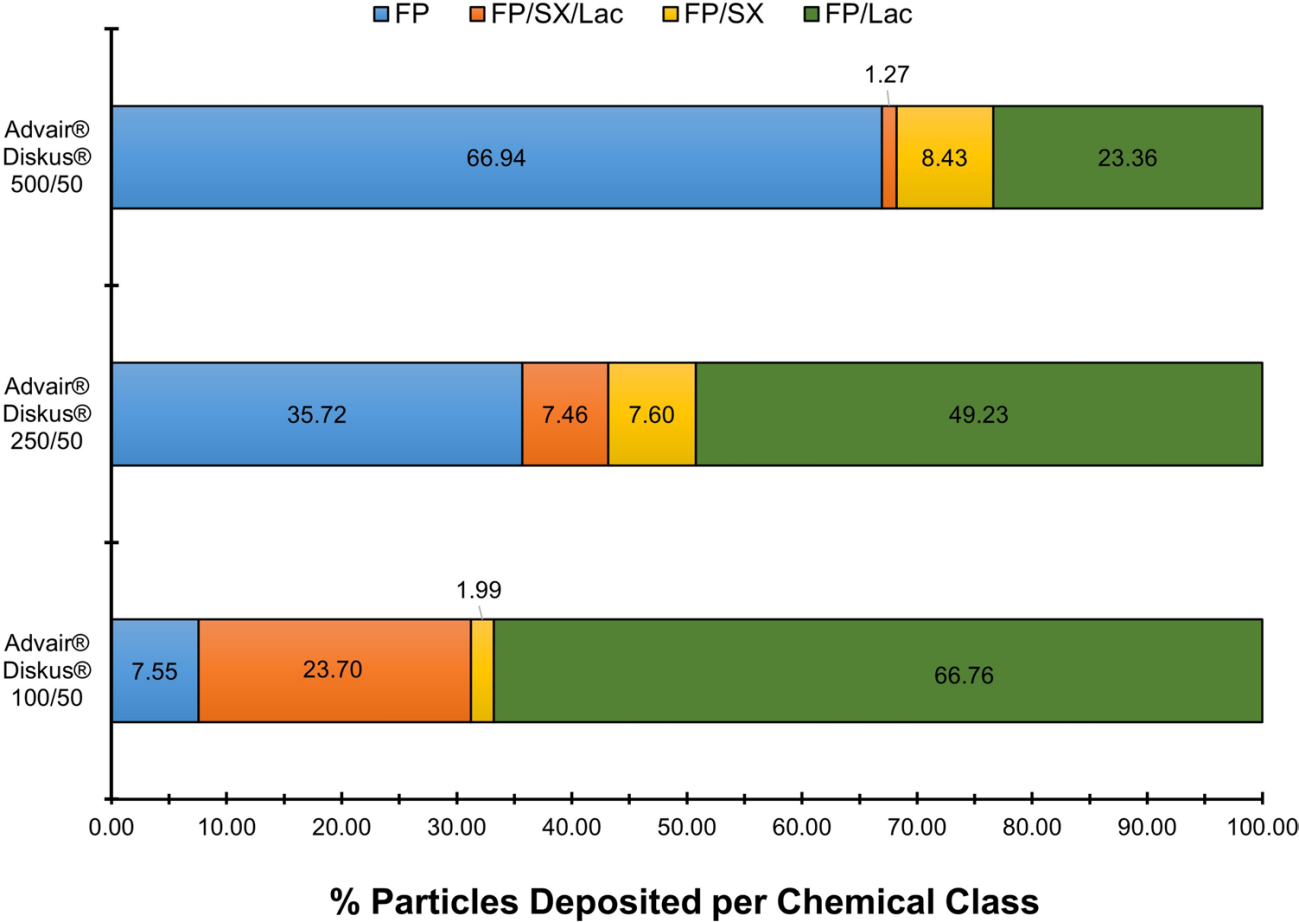
Chemical classification of FP agglomerates of the ISM dose of Advair® Diskus® 100/50, Advair® Diskus® 250/50 and Advair® Diskus® 500/50 DPIs collected at 60 L min^−1^ with a USP inlet and pre-separator connected to the ADC apparatus. Quantities are a percentage by number and an average of 6 independent measurements of at least 3000 particles.

Figure 7 and Table IV shows the microstructural differences following MDRS analysis of samples taken from the ISM collected from Advair® Diskus® 100/50, 250/50 and 500/50 FP/SX (µg/µg) DPI products for FP agglomerates and all the detected agglomerates, respectively. As expected, the data shows the presence of “free-standing” (single particles and agglomerates of a single chemical entity) API and API agglomerated with different components post-aerosolization. The particle size distribution (PSD) of FP for the same products is presented in Table V. The percentage of free-standing FP increased with the greater concentrations of FP in the powder blend while the percentage of free-standing lactose decreases. In addition, the Dv50 of “free-standing” FP increases significantly (ANOVA) suggesting an agglomeration of FP particles on their own. Moreover, the presence of FP/SX/Lac agglomerates particles decreased as the dose strength of FP in Advair® Diskus® increased. A comparison of these data with the dissolution data presented elsewhere (with different batches of Seretide™ Accuhaler™ to the ones herein used) implies that faster dissolving FP in the low dose product may be related to the smaller amount and agglomeration of free-standing FP in those formulations alongside the increased FP agglomeration with more soluble components, Lac and SX, that accelerate FP dissolution [23]. The slower rate of dissolution of FP from both the mid and high-strength formulations was consistent with the greater amount and extent of agglomeration of free-standing FP on its own (Fig. 7) and lower mixed agglomerate structures containing Lac (FP/Lac and FP/SX/Lac), perhaps resulting in poor wettability of FP and a reduction in dissolution rate. FP/SX agglomerates follow the opposite trend. These agglomerates increase as the dose strength of FP in Advair® Diskus® increased, which could be expected to increase the wettability of FP. However, this change appears to have a minimum impact on FP wettability, which could be explained by accounting for a minor change in percentage compared to what is observed for free-standing FP and the lower wettability power of SX compared to Lac due to SX having a comparable particle size to FP and being less soluble than Lac.

**Table IV:** Chemical classification of the ISM dose of Advair® Diskus® 100/50, Advair® Diskus® 250/50, Advair® Diskus® 500/50, Seretide™ Accuhaler™100/50, Flovent® Diskus® 100 and Flixotide™ Accuhaler™ 100 DPIs collected at 60L/min with a USP inlet and pre-separator connected to the ADC apparatus. Quantities are a percentage by number and an average of 6 separate measurements of at least 3000 particles (mean ± standard deviation)

Product	FP	SX	Lac	FP/SX/ Lac	FP/ SX	FP/ Lac	SX/ Lac
Advair® Diskus® 100/50	5.85 ± 0.80	0.79 ± 0.12	18.84 ± 2.46	18.30 ± 7.27	1.53 ± 0.79	51.85 ± 8.73	2.86 ± 2.68
Advair® Diskus® 250/50	34.73 ± 2.73	0.42 ± 0.15	2.05 ± 1.29	7.25 ± 2.86	7.40 ± 2.63	47.84 ± 4.51	0.31 ± 0.34
Advair® Diskus® 500/50	66.75 ± 3.78	0.13 ± 0.06	0.12 ± 0.06	1.26 ± 0.64	8.41 ± 2.02	23.30 ± 1.86	0.02 ± 0.03
Seretide™ Accuhaler™100/50	12.24 ± 2.00	2.25 ± 0.45	18.79 ± 1.56	16.40 ± 2.74	5.72 ± 1.11	41.72 ± 3.88	2.89 ± 0.82
Flovent® Diskus® 100	13.71 ± 2.36	-	40.27 ± 3.40	-	-	46.01 ± 2.85	-
Flixotide™ Accuhaler™ 100	16.89 ± 1.89	-	34.37 ± 5.41	-	-	48.75 ± 4.81	-

**Table V:** The mean particle size in volume distribution of FP particles of Advair® Diskus® 100/50, Advair® Diskus® 250/50, Advair® Diskus® 500/50, Seretide™ Accuhaler™100/50, Flovent® Diskus® 100 and Flixotide™ Accuhaler™ 100 DPIs collected at 60L/min with a USP inlet and pre-separator connected to the ADC apparatus (mean ± standard deviation)

Product	Dv10	Dv50	Dv90	Span
Advair® Diskus® 100/50	2.53 ± 0.26	4.37 ± 0.45	7.32 ± 1.04	1.10 ± 0.17
Advair® Diskus® 250/50	2.79 ± 0.15	4.96 ± 0.25	9.00 ± 0.36	1.25 ± 0.08
Advair® Diskus® 500/50	3.24 ± 0.31	5.86 ± 0.44	10.49 ± 1.17	1.23 ± 0.09
Seretide™ Accuhaler™100/50	2.73 ± 0.37	4.55 ± 0.72	8.81 ± 1.35	1.35 ± 0.27
Flovent® Diskus® 100	2.49 ± 0.22	4.30 ± 0.39	7.15 ± 0.80	1.08 ± 0.05
Flixotide™ Accuhaler™ 100	2.64 ± 0.13	4.39 ± 0.25	7.03 ± 0.38	1.00 ± 0.04

**Fig. 8:**
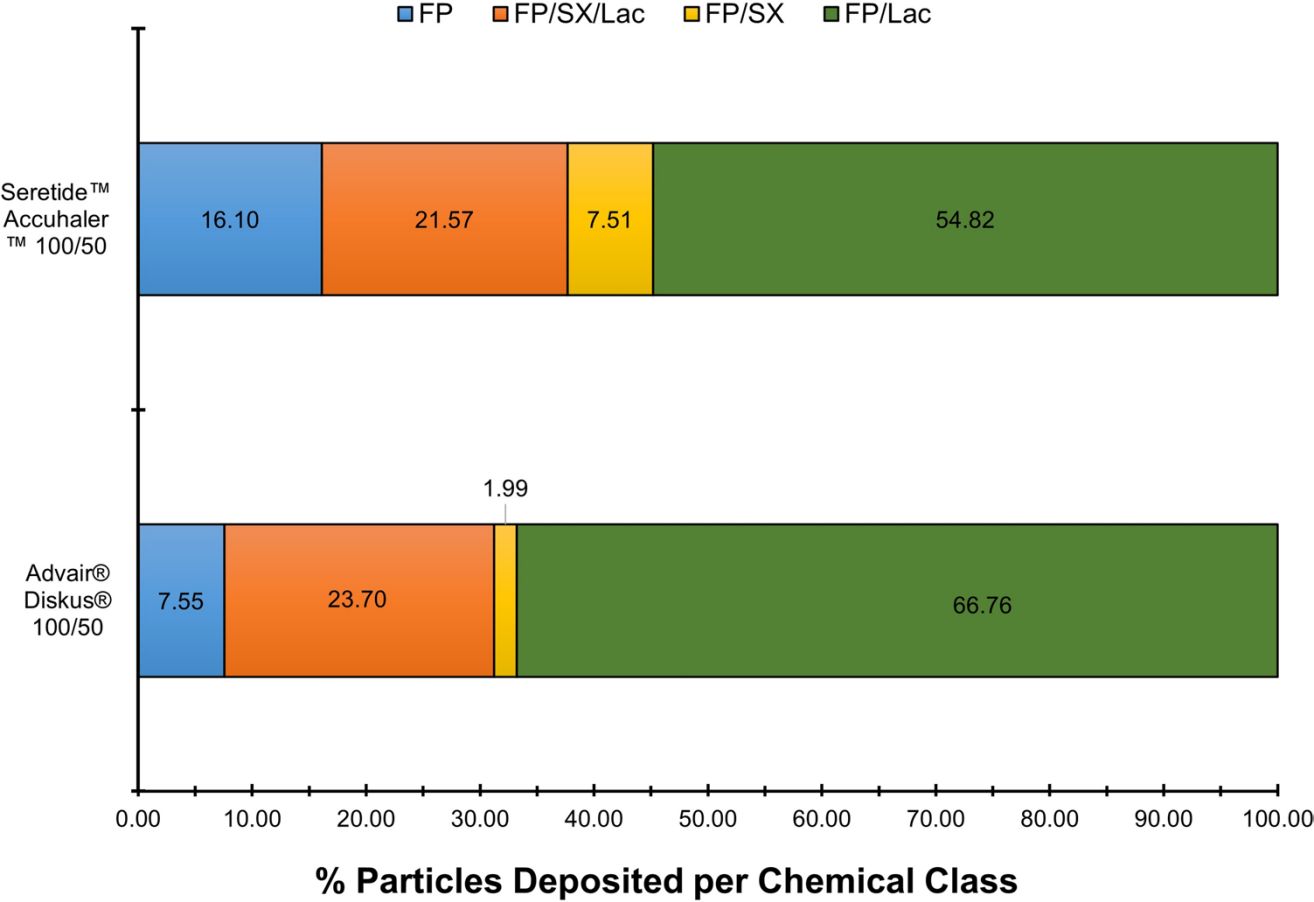
Chemical classification of FP agglomerates of the ISM dose of Advair® Diskus® 100/50 and Seretide™ Accuhaler™100/50 DPIs collected at 60 L min^−1^ with a USP inlet and pre-separator connected to the ADC apparatus. Quantities are a percentage by number and an average of 6 independent measurements of at least 3000 particles.

The state of agglomeration post-aerosolization of FP in combination therapy products with qualitatively and quantitatively equivalence (US Advair® Diskus® 100/50 DPI and Seretide™ Accuhaler™ 100/50 DPI) were also investigated using MDRS. As shown in Fig. 8 and Table IV, Seretide™ Accuhaler™ 100/50 had a larger percentage of free-standing FP compared to Advair® Diskus® 100/50, which had a greater percentage of FP agglomerated with more soluble compounds (Lac and Lac/SX). Thus, the higher dissolution rate of FP observed in Advair® Diskus® could be explained by the larger extent of agglomeration of this low solubility drug (FP) within a highly soluble matrix (Lac and Lac/SX). Once again, the increase in FP/Lac and FP/SX/Lac, and the decrease in free-standing FP appears to outweigh the impact on FP wettability from the observed decrease in FP/SX agglomerates. No significant difference on Dv50 were observed for FP particles of both products.

**Fig. 9:**
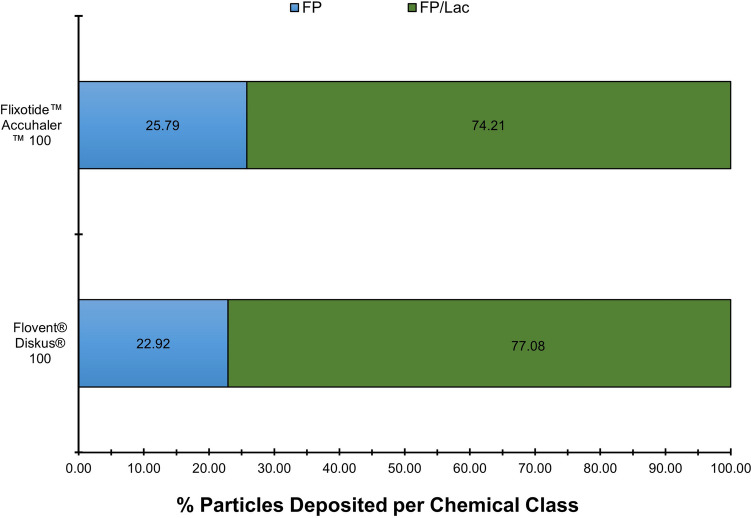
Chemical classification of FP agglomerates of the ISM dose of Flovent® Diskus® 100 and Flixotide™ Accuhaler™ 100 DPIs collected at 60 L min^−1^ with a USP inlet and pre-separator connected to the ADC apparatus. Quantities are a percentage by number and an average of 6 separate measurements of at least 3000 particles.

Figure 9 illustrates state of agglomeration post-aerosolization for the monotherapy products (US Flovent® Diskus® DPI 100 and EU Flixotide™ Accuhaler™ DPI 100). As shown, the percentage of stand-alone FP and its PSD is similar in both products. These results are in agreement with the dissolution data presented earlier.

MDRS has been previously used to investigate the agglomeration and microstructure of different formulations and how it affects formulation performance [14–16]. However, these samples were collected under the nozzles of an impactor that may result in particles depositing in a reduced area and induce an artificial agglomeration of particles [23]. Other techniques such as SPAMS and aerosol time-of-flight mass spectrometry (ATOFMS) have emerged as alternatives to evaluate the agglomeration of dry powder inhalers [36]. Nevertheless, their inability to detect lactose presents a potentially limiting factor understanding how structure impacts performance [36, 37]. Technical advances in MDRS and the development of the ADC apparatus have allowed the analysis of agglomerates in their natural state. One drawback of MDRS is still its ability to only detect particles greater than 0.5 µm; however, the use of an orthogonal technique that is more sensitive to smaller particles such as dissolution might overcome this obstacle. The MDRS data presented here suggests that MDRS and *in* *vitro* dissolution testing could be used as orthogonal techniques in the investigation of low solubility compounds interactive mixtures microstructure and its potential ability link raw material attributes and manufacturing factors to its *in* *vivo* performance.

### Statistical Analysis of Microstructural Evidence

The previous two sections have shown that the rate of dissolution and distribution of agglomerate structures in collected aerosol particles can be used to differentiate FP and FP/SX containing DPI products, FP/SX containing DPI products of different doses, manufacturing location, and between monotherapy and combination products. An ANOVA and Tukey’s honestly significant difference post-hoc test on the percentage of FP dissolved after 2.5 min (the most discriminatory time point) and on MDT (shown in Fig. 4 and Table III) was used to identify statistically significant differences between the products. The dissolution rates of Flovent® Diskus® 100 and Flixotide™ Accuhaler™ 100 are not statistically significantly different (*p* = 0.99 for 2.5 min and *p* = 0.94 for MDT). All other dissolution rates are statistically significantly different (*p* < 0.001 for all for 2.5 min and *p* < 0.05 for MDT), most notably Advair® Diskus® 100/50 and Seretide™ Accuhaler™ 100/50 which, based on their composition, would be expected to behave similarly. These results show the use of dissolution is able to differentiate similar formulations that may have differences in API-excipient agglomeration characteristics or other powder behavior of the aerosolized dose.

**Fig. 10:**
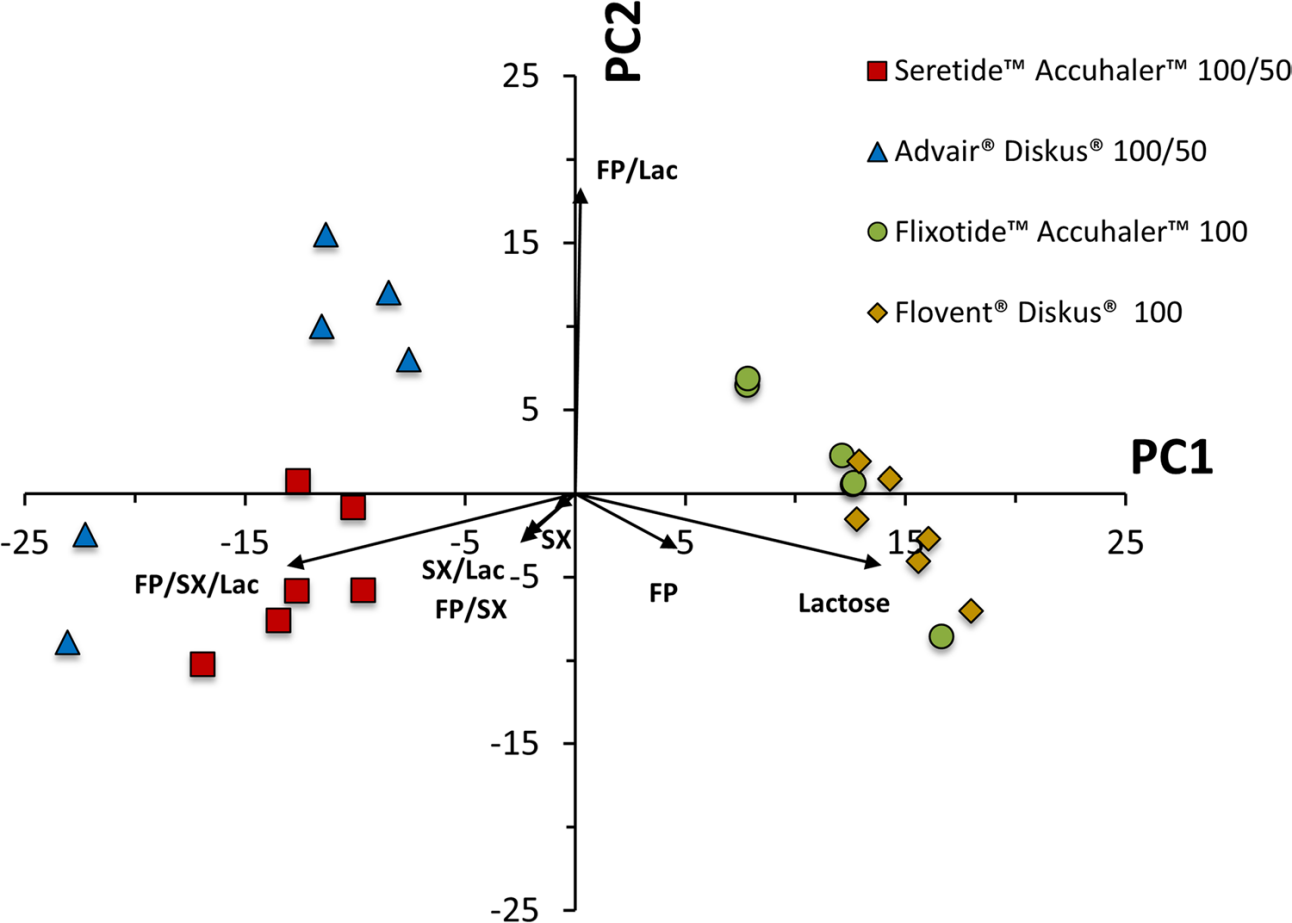
Principal component analysis of MDRS data for Seretide™ Accuhaler™ 100/50, Advair® Diskus® 100/50, Flixotide™ Accuhaler™ 100 and Flovent® Diskus® 100 DPIs. Points show principal component scores for repeat measurements of each formulation and vectors represent the contribution of each chemical class to the principal components.

The MDRS data shown in the previous section produces multi-dimensional results, the interpretation of which depends on the specific product being investigated, and, therefore, requires appropriate treatment. Multivariate statistical analysis, such as principal component analysis (PCA), reduce the number of dimensions of complex datasets and facilitates structural comparisons of the DPIs. In fact, such statistical approaches have already been used with chemical imaging techniques to map the surface of tablets to a high degree of accuracy even when some components are present in vastly smaller quantities than others [38, 39]. PCA creates new uncorrelated variables (principal components) that successively maximize variance. Here PCA was used to identify which agglomerate classes most varied between DPI products. Figure 10 shows the PCA of the individual MDRS measurements from Table 4. The first two principal components, first principal component (PC1, horizontal axis) and second principal component (PC2, vertical axis), account for 93% of the total variance and show good separation of the various DPI products.

The PC1, which accounts for the largest variance, separates the mono and combination products and is composed to a significant extent by the fraction of free-standing FP, free-standing Lac, and FP/SX/Lac. An ANOVA followed by Tukey’s test shows highly statistically significant differences in the fraction of free-standing lactose between the mono and combination products (*p* <  0.001 for all), a somewhat statistically significant difference between the two mono products (*p* = 0.039) and no statistically significant difference in the fraction of free-standing lactose between Advair® Diskus® and Seretide™ Accuhaler™ (*p* = 1.00). MDRS appears to distinguish between mono and combination products, particularly on the agglomeration of FP with lactose and SX and amount of free-standing FP and Lactose.

The PC2, which accounts for the second largest variance, is composed primarily by the fraction of FP/Lac and shows segregation of Advair® Diskus® 100/50 and Seretide™ 100/50. The difference between the fraction of FP/Lac particles between Advair® Diskus® and Seretide™ are statistically significantly different (Welsh’s two-tailed t-test: *p* = 0.036). The segregation of Advair® Diskus® 100/50 and Seretide™ Accuhaler™ 100/50 along the PC2 in Fig. 10 shows a higher proportion of FP/Lac in the Advair® Diskus® particles and a higher proportion of FP agglomerated on its own or with SX in the Seretide™ Accuhaler™ particles. This difference in FP agglomerate structure may explain the statistically significant differences in dissolution kinetics between Advair® Diskus® and Seretide™ Accuhaler™ observed in Fig. 4. Since lactose is more soluble than SX [40, 41], and SX particles are expected to have a similar particle size to FP but smaller than lactose, lactose agglomerated with FP would be expected to dissolve more rapidly and cover a greater FP surface area than SX agglomerated with FP or FP-only agglomerates, resulting in a more rapid increase in wettable surface area, and, therefore, dissolution rate of the FP as discussed above. This correlates well with the dissolution measurements shown in Fig. 4 as FP dissolves faster from Advair® Diskus® than Seretide™ Accuhaler™ and Advair® Diskus® contains a greater quantity of FP/Lac and FP/SX/Lac particles, and lower amount of free-standing FP, as shown in Fig. 8. The combination of MDRS and dissolution has revealed a structural difference in the particles deposited from Advair® Diskus® and Seretide™ Accuhaler™ despite them having similar formulations and manufactured from the same company showing that they are in fact microstructurally dissimilar.

Figure 10 also shows some within batch variability of Advair® Diskus® as two of the Advair® Diskus® samples are located within the Seretide™ Accuhaler™ cluster. It is unknown at this stage whether this between-dose variability is a feature of the drug product or indicative of variation in the MDRS measurement. Therefore, it is necessary to expand the study to multiple batches to identify the key combinations of chemical classes that vary within batch, between-batch, and between-product. After establishing this for a given product, MDRS data could be projected onto the most appropriate axes and combined with dissolution data to compare product similarity. A further advantage of projecting multivariate data onto low-dimensional axes is that a single number can output for incorporation into statistical tests, such as population bioequivalence (PBE), for comparison [42].

## Conclusions

A combination of MDRS and dissolution was utilized to analyze the ISM from various DPI products using a dose collection system, the Aerosol Collection Apparatus (ADC), which deposits the particles uniformly under laminar flow. The key findings in the dissolution studies were that the dissolution rate (taken as the cumulative % drug dissolved after 2.5 min) of particles aerosolized from Seretide™ increased upon a decrease in the nominal FP dose from 500 µg to 100 µg (as reported elsewhere [23]), the mono FP products Flixotide™ Accuhaler™ (EU) and Flovent® Diskus® (US) showed no difference (which would be expected as they contain qualitative and quantitative similar formulations) but, most interestingly, the combination products Seretide™ Accuhaler™ 100/50 (EU) and Advair® Diskus® 100/50 (US) showed different dissolution rate which was unexpected as they too contain similar formulations.

These findings were supported by MDRS data. PCA was used to identify the most appropriate agglomerate classes to categorize the different products when comparing Seretide™ Accuhaler™ 100/50, Advair® Diskus® 100/50, Flixotide™ Accuhaler™ 100 and Flovent® Diskus® 100 DPIs. The fraction of free-standing Lac particles provided the largest difference between mono and combination products. Also, for mono products containing 100 µg of FP, there is a slightly larger percentage of FP-only agglomerates and lower amount of FP agglomerated with other more soluble components which supports the lower FP dissolution rate observed for mono products as compared to the combination products. The fraction of FP agglomerated with Lac provided the largest difference between Advair® Diskus® 100/50 and Seretide™ Accuhaler™ 100/50. Furthermore, a higher proportion of FP agglomerated on its own or with SX could be found on Seretide™ Accuhaler. These findings support the dissolution data as the agglomeration of FP with Lac (large particles freely soluble in water) with or without the presence of SX (FP/Lac and FP/SX/Lac) followed by the agglomeration of FP with SX (similar particle size to FP and sparingly soluble in water) would result in a more rapid increase in FP wettable surface area as the Lac and/or SX dissolve more quickly and, therefore, a faster initial rate of FP dissolution compared to FP-only agglomerates. However, FP agglomerated to Lac appears to be a much more influential factor in the FP dissolution characteristics than FP agglomerated to SX only (FP/SX agglomerates) for these FP/SX combination DPI products.

The *in* *vitro* characterization methods described in this study may provide a novel approach that could aid in supporting the establishment of bioequivalence for generic DPIs with their reference listed drug products or in ensuring consistent batch production, and so requires further study. Further developments would include generating data from a larger number of batches to determine the structural aspects of batch-to-batch variations and evaluating a multiple range of flow rates and/or breathing profiles to assess the impact of patient factors on the deaggregation process. Following this, the orthogonal dissolution and MDRS techniques can be fully combined using multivariate methods and a methodology for designing a combination of structural data that may be able to serve as a product-specific parameter for use in statistical tests, such as population bioequivalence (PBE).
